# Mortality prediction after major surgery in a mixed population through machine learning: a multi‐objective symbolic regression approach

**DOI:** 10.1111/anae.16538

**Published:** 2025-01-08

**Authors:** Pietro Arina, Davide Ferrari, Nicholas Tetlow, Amy Dewar, Robert Stephens, Daniel Martin, Ramani Moonesinghe, Vasa Curcin, Mervyn Singer, John Whittle, Evangelos B. Mazomenos

**Affiliations:** ^1^ Bloomsbury Institute of Intensive Care Medicine University College London London UK; ^2^ Human Physiology and Performance Laboratory, Centre for Perioperative Medicine, Research Department of Targeted Intervention University College London London UK; ^3^ Peninsula Medical School University of Plymouth Plymouth Devon; ^4^ Department of Population Health Sciences King's College London London UK; ^5^ Department of Medical Physics and Biomedical Engineering University College London London UK; ^6^ Wellcome/Engineering and Physical Sciences Research Council Centre of Interventional and Surgical Sciences London UK

**Keywords:** cardiopulmonary exercise testing, machine learning, mortality, multi‐objective symbolic regression

## Abstract

**Introduction:**

Understanding 1‐year mortality following major surgery offers valuable insights into patient outcomes and the quality of peri‐operative care. Few models exist that predict 1‐year mortality accurately. This study aimed to develop a predictive model for 1‐year mortality in patients undergoing complex non‐cardiac surgery using a novel machine‐learning technique called multi‐objective symbolic regression.

**Methods:**

A single‐institution database of patients undergoing major elective surgery with previous cardiopulmonary exercise testing was divided into three datasets: pre‐operative clinical data; cardiorespiratory and physiological data; and combined. A multi‐objective symbolic regression model was developed and compared against existing models. Model performance was evaluated using the F1 score. Shapley additive explanations analysis was used to identify the major contributors to model performance.

**Results:**

From 2145 patients in the database, 1190 were included, with 952 in the training dataset and 238 in the test dataset. Median (IQR [range]) age was 71 (61–79 [45–89]) years and 825 (69%) were male. The multi‐objective symbolic regression model demonstrated robust consistency with an F1 score of 0.712. Shapley additive explanations analysis indicated that ventilatory equivalents for carbon dioxide, oxygen at peak exercise and BMI influenced model performance most significantly, surpassing surgery type and named comorbidities.

**Discussion:**

This study confirms the feasibility of developing a multi‐objective symbolic regression‐based model for predicting 1‐year postoperative mortality in a mixed non‐cardiac surgical population. The model's strong performance underscores the critical role of physiological data, particularly cardiorespiratory fitness, in surgical risk assessment and emphasises the importance of pre‐operative optimisation to identify and manage high‐risk patients. The multi‐objective symbolic regression model demonstrated high sensitivity and a good F1 score, highlighting its potential as an effective tool for peri‐operative risk prediction.

## Introduction

Peri‐operative medicine encompasses the full range of care, from initial contemplation of surgery to final recovery [1]. Postoperative mortality is influenced by patient comorbidities and the quality of peri‐operative care. Globally, the 1‐year postoperative mortality rate is approximately 5–6% [2, 3, 4, 5, 6, 7, 8, 9]. Postoperative mortality ranks as the third leading cause of death worldwide [8, 9]. As the surgical population becomes older and more vulnerable, the risk of peri‐operative mortality increases [10]. One‐year mortality rates for patients with frailty undergoing major surgery are approximately 14%, nearly triple that of patients who are not frail [6].

Accurate evaluation of peri‐operative risk is thus crucial for informed clinical decision‐making [11, 12, 13]. Most peri‐operative risk prediction models focus on in‐hospital or 30‐day postoperative outcomes; however, these have limitations. Since these outcome measures were developed primarily to audit surgical practice, they cannot provide insight into the evolution of outcomes in the months and years following surgery [14, 15]. To address this limitation, new longer‐term prediction models should be developed. These would offer a broader appreciation of long‐term health outcomes, potentially including complications and health‐related quality of life that are not captured by current models. This extended timeframe can support shared decision‐making, enabling clinicians and patients to weigh the risks and benefits of surgery compared with alternative treatments, offering a more comprehensive view of prognosis and guiding choices that align with the patient's long‐term health goals and expectations [14, 16]. A 1‐year mortality model after surgery could be compared against an individual's predicted 1‐year survival without surgery, balancing the surgical risk against the natural progression of the patient's comorbidities [15, 17]. Currently, few studies predict 1‐ to 5‐year mortality risk in non‐cardiac surgery [17].

Large population studies have shown that cardiorespiratory fitness is the strongest predictor of mid‐ to long‐term morbidity and mortality in adults [18]. Cardiopulmonary exercise testing (CPET) is well established as the gold standard for assessing cardiorespiratory fitness in at‐risk patients before major surgery [19, 20]. It provides a dynamic, individualised assessment of physiology under standardised stress and is useful for predicting postoperative morbidity and mortality across various types of surgery [21, 22, 23, 24]. Notably, patients undergoing major surgery for oncological diseases are more likely to die from cardiovascular complications than from the primary cancer itself [25].

Substantial research has focused on using machine learning to predict peri‐operative mortality [17]. However, forecasting postoperative mortality accurately with machine learning is challenging, as highlighted by recent studies reporting low F1 scores with high area‐under‐the‐curve values. This reflects limitations in predictive accuracy due to unbalanced datasets where certain outcomes or classes are underrepresented, leading to biased predictions and insufficient feature representation [17, 26].

To overcome these limitations, multi‐objective symbolic regression may be used. This is a technique based on genetic programming that formulates a series of comprehensible mathematical equations to formulate predictive models [27, 28, 29]. It has shown effectiveness in managing unbalanced datasets and creating models sensitive to specific data characteristics [27, 28]. By employing multi‐objective symbolic regression, we address the challenges of classical machine learning, focusing particularly on dataset imbalance.

In this study, we aimed to develop a 1‐year mortality model for patients undergoing high‐complexity, non‐cardiac surgery using multi‐objective symbolic regression trained on pre‐operative clinical, cardiorespiratory and physiological data. Additionally, we explored the relative importance of fitness features using Shapley additive explanation (SHAP) analysis to enhance our understanding of how various factors correlate with mortality and to provide insights into peri‐operative care.

## Methods

The study was conducted in accordance with TRIPOD+AI guidelines [30]. University College London Hospitals NHS Foundation Trust maintains a prospective research database of patients undergoing CPET before major complex surgery. All participants provided written consent for their CPET outcomes to be included in the database for future research, in compliance with the Declaration of Helsinki. Ethical approval was initially granted in 2012 and reaffirmed in 2019, with no specified time constraints. The database contains data for assessing and studying short‐ and long‐term postoperative morbidity and mortality, encompassing a broad range of patients who underwent CPET before complex major surgery. The database was queried for patients enrolled between 2012 and 2022. Patients aged ≥ 18 y referred for pre‐operative assessment and scheduled for elective surgery were included. Those aged < 18 y or incapable of providing informed consent were not included.

The dataset is organised into two principal feature categories, along with recorded outcomes. These are detailed comprehensively in online Supporting Information Appendix S1. The clinical dataset includes 39 parameters, including patient characteristics; medical history; laboratory test results; and specifics of the surgical procedure. The cardiorespiratory fitness dataset had 46 parameters including oxygen consumption (V̇O_2_); carbon dioxide production (V̇CO_2_); end‐tidal gas composition (P_ET_O_2_ and P_ET_CO_2_) during a protocolised exercise test on a cycle ergometer; ventilatory equivalents for oxygen (VE.V̇O_2_^‐1^) and carbon dioxide (VE.V̇CO_2_^‐1^); oxygen pulse (V̇O_2_.HR^‐1^); peak oxygen consumption rate (V̇O_2_ peak); and the ventilatory anaerobic threshold established through the V‐slope method [19]. Peak V̇O_2_ and anaerobic threshold values were indexed to body weight (ml.min^‐1^.kg^‐1^). Electrocardiographic and expired gas data were collected at 1‐s intervals, with median filtering. Initial analysis was carried out by two clinical exercise physiologists and further validated by a consultant anaesthetist. Outcomes and clinical scores were collected, including ASA physical status [31]; Duke Activity Status Index (DASI) score [32]; Portsmouth‐Physiology and Operative Severity Score for the Enumeration of Mortality and Morbidity (P‐POSSUM) [33]; postoperative care destination; duration of hospital stay; and mortality at 30 days and one year. One‐year mortality was prospectively assessed by using hospital electronic health records (from 2019 onwards; EPIC, Verona, WI, USA), with additional data sourced from general practitioners through telephone and email communication. Deaths resulting from acute, traumatic events (e.g. car accidents or violence) were not included. The study adhered to the Peri‐operative Exercise Testing and Training Society's guidelines for conducting CPET [19]. Clinical laboratory results obtained in the pre‐operative period were obtained from the hospital electronic health record (EPIC) for data from 2019 onwards, while data before 2019 were recorded by hand by research nurses in the patient research log. Data were stored in read‐only Microsoft Excel (Microsoft Corporation, Redmond, WA, USA) databases located on secure university servers. Dataset integrity and quality were checked by three physicians independently.

Analyses were performed using Python (version 3.10.12) [34] and Pandas (version 1.4.2) [35]. Patients with incomplete data were excluded from analyses. For categorical variables, binary encoding was implemented with a value of 1 indicating the presence of an event and 0 indicating absence. Attributes such as sex, type of surgery and surgical specialty were converted using one‐hot encoding to address their non‐ordinal characteristics [36]. Continuous variables were scaled to a range from 0 to 1. Categorical data were analysed through frequency distributions. Differences between groups were analysed using either Student's t‐test or the Mann–Whitney U test, depending on the data distribution. A standard Cox proportional hazards analysis was conducted to examine associations between model features and 1‐year mortality and a sensitivity analysis, using a Kruskal‐Wallis test, was performed to compare outcomes across different time periods [37].

Power analysis revealed that a sample size of at least 1000 patients would be adequate to develop and validate a 1‐year mortality prediction model with a mortality rate of 5.5% at a 5% significance level and 80% power. Creating a peri‐operative 30‐day mortality model for comparison was unfeasible as the 30‐day mortality rate of 1.9% meant the available sample size lacked sufficient statistical power.

The relative rarity of mortality at 1 year resulted in an unbalanced dataset where deceased patients were underrepresented, hampering machine learning algorithms. To address this, we chose multi‐objective symbolic regression, a highly adaptable and effective machine learning algorithm suited for handling unbalanced datasets [27, 28, 29, 38]. Utilising genetic programming to derive mathematical formulae for learning tasks, multi‐objective symbolic regression combines a range of mathematical operations, from simple to complex, into a learnable model without a predefined structure [28]. Multi‐objective symbolic regression's automated feature selection during training is particularly beneficial for unbalanced datasets, prompting inclusion of all available variables into the analyses. We developed and refined 300 unique models over 500 generations, optimising binary cross‐entropy and F1 score to balance false negatives and false positives.

To compare the capabilities of multi‐objective symbolic regression on the same task, we trained models using the PyCaret Python library [39]. The final multi‐objective symbolic regression models are provided in online Supporting Information Appendix S1.

Models were assessed using classification metrics such as accuracy; sensitivity; specificity; F1 score; positive predictive value; negative predictive value; and area under the curve values [38]. Calibration plots are reported in online Supporting Information Figure S1. To compare the models, we focused on the F1 score, the harmonic mean of precision and recall [40]. The F1 score is more relevant than accuracy in this study as it emphasises false positives and false negatives. While accuracy is suited for balanced classes and when true positives and true negatives are key, the F1 score is better for imbalanced classes, as in this study [41].

The decision to divide the experiments into a clinical data set, cardiorespiratory and physiology dataset and a combined full dataset was made to assess the individual contributions of each segment to the models, as well as their collective impact.

The dataset was divided into training and testing subsets using an 80/20 split, ensuring consistent outcome prevalence representation [42]. Hyperparameter tuning was achieved via a grid search 10‐fold cross‐validation on the training dataset, adhering to a 90/10 division. To evaluate the predictive performance and consistency of each model, 10 separate test runs were conducted on a 90% portion of the test dataset selected randomly, averaging the performance results obtained. Finally, an in‐depth error analysis was conducted on data points predicted incorrectly by the machine learning models to understand between‐class clinical distinctions.

Finally, SHAP analysis was applied to the best‐performing multi‐objective symbolic regression model to assess feature importance on outcomes [43]. This model was chosen based on superior sensitivity, specificity and area under the curve values. Shapley additive explanation is a model‐agnostic tool, evaluating feature importance exclusively and offering insights into clinical data correlations.

## Results

**Figure 1:**
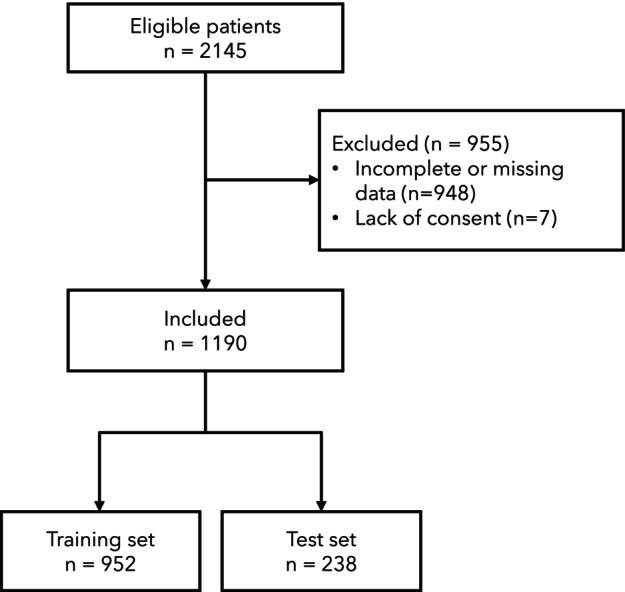
Patient flow diagram.

From a total of 2145 patients in the database, 1190 with complete data were included (Fig. 1). Patient characteristics are reported in Table 1 and online Supporting Information Table S1. The ethnic distribution was congruous with that of the UK population reported in the 2021 census [44].

**Table 1:** Patient and procedure characteristics, cardiopulmonary exercise testing values, and outcomes. Values are median (IQR [range]) or number (proportion).

	Full dataset	Training set	Test set	p value
	n = 1190	n = 952	n = 238	
Age; y	71 (61–79 [45–89])	71 (61–79 [45–89])	73 (59–79 [49–88])	0.88
Sex; male	825 (69%)	653 (69%)	166 (70%)	0.81
BMI; kg.m^‐2^	26.5 (23.3–30.0 [14.0–39.0])	26.4 (23.1–30.0 [16.0–39.0])	26.0 (23.4–29.4 [14.0–38.0])	0.30
ASA physical status	2 (2–3 [0–4])	2 (2–3 [0–4])	2 (2–3 [1–4])	0.23
Duke Activity Status Index	46.2 (32.2–58.2 [31.4–58.2])	45.4 (31.4–58.2 [31.4–58.2])	42.7 (32.2–58.2 [31.4–58.2])	0.50
**Ethnicity**				
Asian	60 (5%)	48 (5%)	12 (5%)	0.67
Arabic	31 (3%)	24 (3%)	7 (3%)	0.56
Black	78 (7%)	62 (7%)	16 (7%)	0.45
Mixed/other	56 (5%)	44 (5%)	12 (5%)	0.65
White	966 (81%)	776 (82%)	193 (80%)	0.76
**Comorbidities**				
Hypertension	416 (35%)	332 (35%)	84 (35%)	0.88
Diabetes	131 (11%)	109 (11%)	22 (11%)	0.40
Angina	48 (4%)	34 (3%)	14 (5%)	0.30
Coronary stent	60 (5%)	50 (5%)	10 (4%)	0.53
Coronary artery bypass graft	36 (3%)	24 (2%)	12 (3%)	0.25
Chronic cardiac failure	119 (10%)	93 (10%)	26 (11%)	0.83
Peripheral vascular disease	24 (2%)	19 (2%)	5 (2%)	0.96
CVA or TIA	47 (4%)	38 (4%)	9 (3%)	0.62
COPD	72 (6%)	62 (6%)	10 (4%)	0.18
Asthma	95 (8%)	73 (7%)	22 (8%)	0.68
Pulmonary embolism	17 (2%)	13 (2%)	4 (2%)	0.88
Pulmonary fibrosis	10 (1%)	6 (1%)	4 (2%)	0.16
Smoking (ex/current)	427 (36%)	335 (36%)	92 (38%)	0.36
**Medications**				
Beta blocker	238 (20%)	191 (20%)	47 (20%)	0.51
Nitrates	36 (3%)	29 (3%)	7 (4%)	0.84
ACE inhibitors	214 (18%)	166 (17%)	48 (19%)	0.64
Statins	357 (30%)	282 (30%)	76 (31%)	0.81
**Surgical specialty**				
Colorectal	244 (20%)	196 (20%)	48 (20%)	0.80
Upper gastrointestinal	196 (17%)	155 (17%)	41 (17%)	0.80
Genito‐urinary	404 (34%)	322 (34%)	82 (34%)	0.80
Head and neck	253 (21%)	201 (21%)	52 (21%)	0.80
Thoracic	43 (4%)	33 (4%)	10 (4%)	0.80
Others	50 (4%)	30 (4%)	10 (4%)	0.80
**Cardiopulmonary exercise testing values**				
Metabolic equivalents	4.5 (3.7–5.5 [1.47–10.7])	4.5 (3.7–5.5 [1.56–10.7])	4.5 (3.7–5.5 [1.47–9.8])	0.83
Anaerobic threshold; V̇O_2_.kg^‐1^ ml.kg^‐1^.min^‐1^	10.5 (9.0–12.4 [2.9–25.0])	10.6 (9.0–12.4 [3.4–25.0])	10.5 (9.2–12.4 [2.9–18.0])	0.64
Peak V̇O_2_.kg^‐1^; ml.kg^‐1^.min^‐1^	17.0 (14.0–20.5 [8.2–46.0])	17.0 (13.8–20.5 [9.2–46.0])	16.8 (14.2–20.6 [8.2–42.0])	0.61
Peak VE.V̇CO_2_^‐1^; ml.min^‐1^	34.5 (31.4–38.8 [17.5–56.0])	34.6 (31.4–38.7 [17.5–49.0])	34.8 (31.2–38.8 [19.4–56.0])	0.98
**Outcomes**				
1‐year mortality	66 (6%)	52 (5%)	14 (6%)	0.86
30‐day mortality	23 (2%)	18 (2%)	5 (2%)	0.56
Readmission 30 days	88 (7%)	70 (7%)	18 (8%)	0.67
Adverse event	178 (15%)	142 (15%)	36 (15%)	0.76
Postoperative location; ward/PACU/ICU	345/762/83 (29%/64%/7%)	276/610/66 (29%/64%/7%)	69/152/17 (29%/64%/7%)	0.65
Duration of hospital stay; days	10 (7–16 [2–52])	10 (7–16 [2–42])	10 (7–16 [4–52])	0.65

CVA, cerebrovascular accident; TIA, transient ischaemic attack; COPD, chronic obstructive pulmonary disease; ACE, angiotensin‐converting enzyme; V̇O_2_.kg^‐1^, oxygen consumption per kilo; VE.V̇CO_2_^‐1^, ventilatory efficiency/carbon dioxide output; PACU, postanaesthetic care unit.

In the context of predicting 1‐year mortality, when the full dataset was used, multi‐objective symbolic regression achieved the highest F1 score (0.712) and sensitivity (0.911) compared with other models. In the fitness dataset alone, multi‐objective symbolic regression showed a sensitivity of 0.447 and an F1 score of 0.343, both of which were higher than those of the other models, except for the support vector machine classifier, which showed an accuracy of 0.457 and an F1 score of 0.317. The analysis of test vs. training set performance showed that the multi‐objective symbolic regression model exhibited consistency, with the F1 score in the test set (0.712) closely matching that in the training set (0.725), indicating minimal performance degradation. In contrast, all other models showed significant performance drops in the test set compared with the training set. For example, the Ada‐Boost classifier had an F1 score of 0.847 in the training set and 0.386 in the test set, suggesting overfitting. Multi‐objective symbolic regression showed robust generalisation capabilities, performing consistently across different datasets and achieving a balanced performance across precision, sensitivity and specificity, supporting its ability to manage both false positives and false negatives effectively.

**Figure 2:**
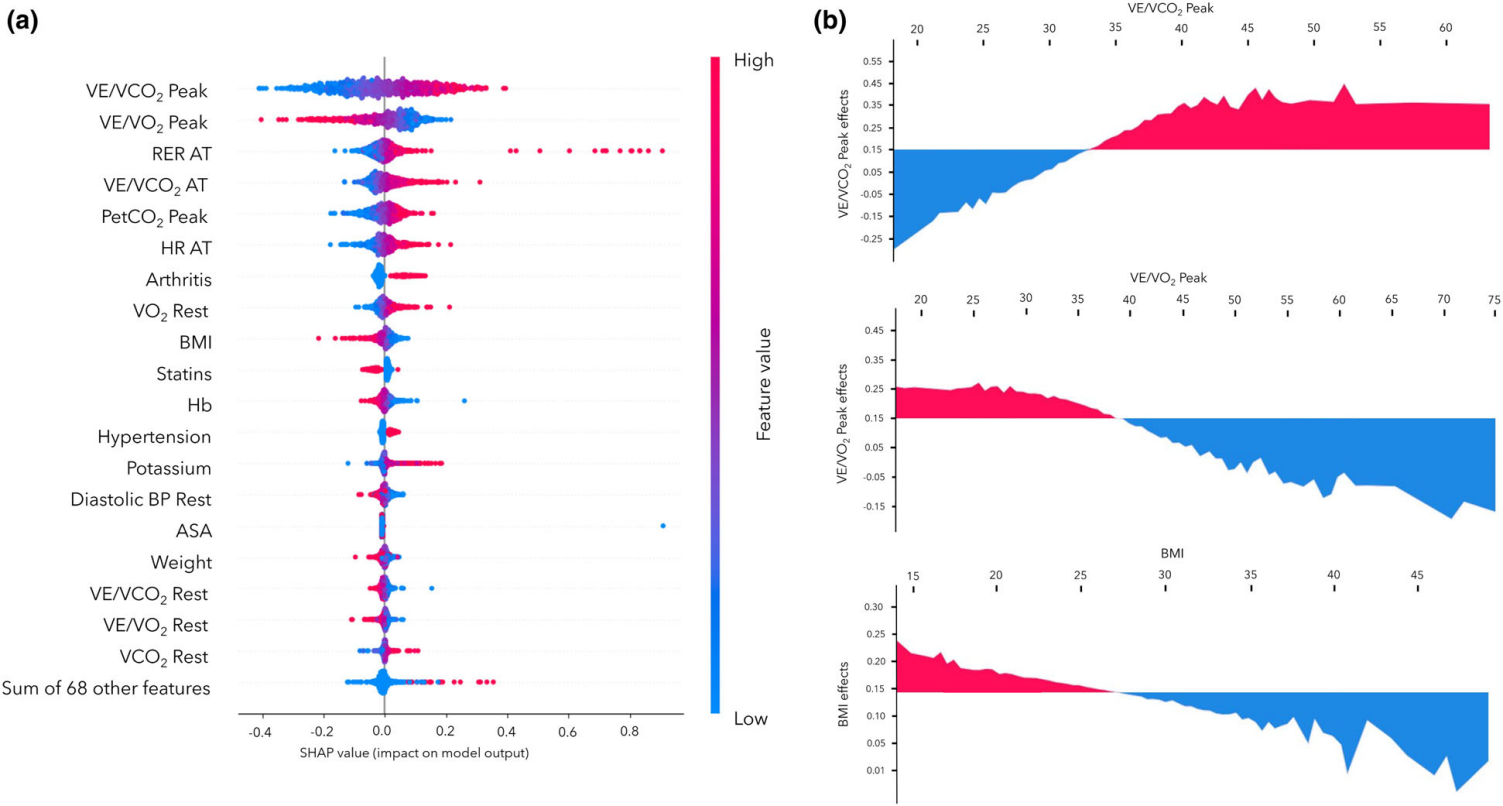
(a) Shapley additive explanations (SHAP) analysis for a multi‐objective symbolic regression model. (b) Graphs analysing the influence of specific respiratory and physiological variables on a predictive model. VE.V̇CO_2_^‐1^, ventilatory efficiency/carbon dioxide output; VE.V̇O_2_^‐1^, ventilatory efficiency/oxygen consumption; RER, respiratory equivalent ratio; AT, anaerobic threshold; PetCO_2_, partial pressure of end‐tidal carbon dioxide; HR, heart rate; V̇O_2_, oxygen consumption; Hb, haemoglobin; BP, blood pressure; V̇CO_2_, carbon dioxide output.

The SHAP values for the multi‐objective symbolic regression model applied to peri‐operative mortality prediction are shown in Fig. 2. The SHAP analysis highlighted the clinical significance of different features in predicting mortality risk. Higher peak VE.V̇CO_2_^‐1^ was associated with an increased risk of mortality. Conversely, BMI values > 28 kg.m^‐2^ were linked to a lower risk of 1‐year mortality. VE.V̇O_2_^‐1^ values > 38 ml.min^‐1^ at both peak and rest (indicative of better aerobic capacity) were associated with lower SHAP values, indicating a lower risk of 1‐year mortality.

Across all models, the distributions of peak VE.V̇CO_2_^‐1^, age and BMI for false positives and false negatives were notably similar, indicating that errors are more likely due to inherent difficulties in predicting certain patient profiles from clinical data rather than specific deficiencies in the machine learning algorithms (online Supporting Information Figure S2).

Cox proportional hazards analysis (online Supporting Information Table S2) indicated that the presence of previous myocardial ischaemia and VE.V̇CO_2_^‐1^ at anaerobic threshold and peak were associated with an increased 1‐year mortality risk (hazard ratio (95%CI) 1.15 (1.12–1.83) and 1.17 (1.09–1.25), respectively). Sensitivity analysis revealed no difference in outcomes when comparing patient results over time. Results of the machine learning models used to predict 1‐year mortality are in online Supporting Information Table S3.

## Discussion

This study showed the feasibility of creating a 1‐year mortality model for patients undergoing complex non‐cardiac surgery using multi‐objective symbolic regression, utilising pre‐operative data from both clinical and fitness domains. The model proved effective in classifying patients despite an unbalanced dataset, exhibiting high sensitivity, a high F1 score and no overfitting. We have shown that longer‐term outcomes are influenced by several interacting factors, including physiological reserve and the progression of underlying comorbidities.

Accurate prediction of 1‐year mortality is useful for evaluating patients undergoing elective major surgery due to its significant ethical and clinical implications for decision‐making. One‐year mortality is an underused outcome that can reflect underlying disease or age‐related mortality risk with or without surgery [15]. As cardiorespiratory fitness is an independent risk factor for 1‐year mortality [18], incorporating CPET data enhances the model's impact further by accounting for the influence of physiological age or reserve. Apart from providing the peri‐operative care team with information to aid selection of the most appropriate surgical approach, it could also help identify patients for pre‐operative prehabilitation and optimisation pathways, with the potential to improve outcomes [45].

A 1‐year mortality model complements existing peri‐operative models that predict in‐hospital mortality or 30‐day postoperative outcomes primarily. Such models include the pre‐operative mortality predictor and the universal American College of Surgeons National Surgical Quality Improvement Program Surgical Risk Calculator that now incorporates machine learning techniques [46, 47]. These models rely mainly on patient characteristics and type of surgery but do not integrate any assessment of cardiorespiratory fitness. The ASA physical status for example, though used widely, relies heavily on subjective clinician assessment [46].

Our findings emphasise the significance of both clinical history and physiological assessment (cardiorespiratory fitness) in predicting longer‐term health outcomes. The SHAP analysis of the multi‐objective symbolic regression model highlights the importance of physiological metrics obtained from pre‐operative CPET (particularly VE.V̇CO_2_^‐1^) in forecasting peri‐operative mortality, aligning with the existing literature [19, 48, 49, 50]. The VE.V̇CO_2_^‐1^ ratio is a correlate of ventilation/perfusion matching [51] and has been related to cardiac output and mortality risk in patients with heart failure [52], as well as the development of early postoperative morbidity and mortality [52].

Body mass index was also identified as a critical predictor. A high BMI often indicates patients who are overweight or living with obesity, which is associated commonly with heart disease, metabolic syndrome and diabetes. Our model, however, suggested a protective influence of elevated BMI and a detrimental influence of low BMI, potentially representative of sarcopenia, cachexia and other chronic health conditions [53, 54]. This relationship is termed the ‘obesity paradox’ where, in certain populations and conditions, individuals who are overweight or obese have better survival outcomes compared with those with a normal BMI. This phenomenon has been particularly noted in patients with chronic diseases such as chronic kidney disease and certain types of cancer [55, 56]. This concept is, however, controversial [57]. Patients with a high BMI may have higher intrinsic metabolic reserves to cope with critical and/or chronic illness and potentially protective adipose tissue cytokines. On the other hand, patients with chronic diseases may simply reflect a sicker, more frail population, which are risk factors in themselves. Body mass index also does not distinguish between muscle and fat mass.

The multi‐objective symbolic regression model exhibited minimal overfitting, maintaining consistent performance between the training and testing sets. The other models examined showed significant performance degradation when applied to the test set, suggesting potential overfitting. In predictive modelling for clinical applications where data can be highly unbalanced, traditional metrics such as accuracy and area under the curve values can be misleading; the F1 score is a more critical measure. The multi‐objective symbolic regression model achieved a commendable F1 score, emphasising its suitability for this complex problem.

Using the full database, the multi‐objective symbolic regression model showed high sensitivity, improving identification of high‐risk patients. This minimises false negatives, which is crucial in clinical settings where missing a high‐risk patient could have significant consequences. Such patients can be directed towards peri‐operative prehabilitation or medical optimisation pathways, potentially enhancing their outcomes, though we acknowledge there will be a higher rate of false positives who may not benefit from such interventions. Multi‐objective symbolic regression presents an alternative to traditional predictive models, which often suffer from technical constraints and a narrow selection of features leading to suboptimal performance, especially in the face of unbalanced datasets that are commonplace in medical data [17]. Studies should be conducted to elucidate mechanisms underlying the relationships between cardiorespiratory fitness and peri‐operative outcomes, with a view to identifying targetable mechanisms for intervention. Furthermore, external databases should be identified to enable external validation of the model for broader generalisability.

This study has several limitations. It was conducted at a single institution, but the 1‐year mortality in our database is congruent with that reported in the literature, thus improving the likely generalisability of our findings [2]. External validation with independent datasets from other institutions would be necessary to confirm robustness and applicability in different populations and settings. The dataset contained a White majority population, meaning the results should be interpreted with caution and may not apply to other ethnicities [2]. Another limitation pertains to the unavailability of data on patients' body composition and investigation of the muscle‐to‐adipose tissue ratio. There is also a potential for selection bias since patients referred for CPET may represent a subset with higher perceived risk, potentially skewing results. This is somewhat mitigated in our database since CPET is a routine component of most of our surgical pathways. The use of retrospective data can also introduce bias relating to data completeness and quality. We employed a three‐researcher data quality check to mitigate against this. Unmeasured confounders include socio‐economic status, nutritional status and other unrecorded comorbidities. Rapid advancements in machine learning and genetic programming may lead to newer, more sophisticated methods that could outpace multi‐objective symbolic regression, necessitating ongoing research and comparison with emerging techniques. While our model does appear to perform well, its practical utility in clinical decision‐making needs evaluation. This would include addressing how well the model integrates into existing clinical workflows and its impact on management strategies and patient outcomes.

In conclusion, we generated a 1‐year mortality model for patients undergoing major complex surgery that emphasises the need to consider both demographic and fitness factors. The ability of multi‐objective symbolic regression to handle complex data sets the stage for future validation studies and, potentially, integration of such models into clinical practice.

## Supporting information


**Figure S1.** Calibration plots for various machine learning models.
**Figure S2.** Box plots showing the distribution of key variables.

**Table S1.** Patient and procedure characteristics and outcomes.
**Table S2.** Relationship between clinical features and 1‐year mortality.
**Table S3.** Performance of different machine learning models.

**Appendix S1.** Model information.
