# Supplementary material for: Mortality prediction after major surgery in a mixed population through machine learning: a multi‐objective symbolic regression approach
Source: Anaesthesia. 2025 Jan 8;80(5):551–60. doi: 10.1111/anae.16538 (PMC7617356; doi:10.1111/anae.16538)
Supplement: Supplementary file 1 — Figure S1. Calibration plots for various machine learning models. Figure S2. Box plots showing the distribution of key variables. [file ANAE-80-551-s001.docx]

**Figure S1.** Calibration plots for various machine learning models, including logistic regression (LR); Extreme Gradient Boosting (XGBoost); AdaBoost (ADA); support vector machines (SVM); k-nearest neighbours (KNN); and Multi-Objective Symbolic Regression (MOSR) applied to the fitness (CRF), clinical and full datasets. These plots illustrate the alignment between predicted and observed outcomes, providing insight into each model's predictive performance across different datasets.

**Figure S2.** Box plots showing the distribution of three key variables—ventilatory efficiency/carbon dioxide output at Peak (VE/V̇CO2 peak), age, and body mass index (BMI)—across different error types in three predictive models: Multi-Objective Symbolic Regression (MOSR), AdaBoost (ADA), and support vector machine (SVM). Each row of box plots corresponds to one variable, with plots organised by model and error type, including true negative (TN), false positive (FP), false negative (FN), and true positive (TP).
