# Supplementary material for: Mortality prediction after major surgery in a mixed population through machine learning: a multi‐objective symbolic regression approach
Source: Anaesthesia. 2025 Jan 8;80(5):551–60. doi: 10.1111/anae.16538 (PMC7617356; doi:10.1111/anae.16538)
Supplement: Supplementary file 2 — Table S1. Patient and procedure characteristics and outcomes. Table S2. Relationship between clinical features and 1‐year mortality. Table S3. Performance of different machine learning models. [file ANAE-80-551-s003.docx]

**Table S1.** Patient and procedure characteristics, cardiopulmonary exercise testing (CPET) values and outcomes. Values are median (IQR [range]) or number (proportion).

|  | **Full dataset**  **n = 1190** | **Training set**  **n = 952** | **Test set**  **n = 238** | **p value** |
| --- | --- | --- | --- | --- |
| P-POSSUM; operative | 14 (12–16 [10–18]) | 14 (11–16 [10–18]) | 14 (12–16 []10–18) | 0.45 |
| P-POSSUM; physiological | 18 (16–21 [12–21]) | 18 (15–21 [12–21]) | 18 (16–21 [12–21]) | 0.43 |
| **Laboratory values** | | | | |
| Na meq.l^-1^ | 141 (139–142 [126–152]) | 141 (139–143 [126–151]) | 141 (138–142 [128–152]) | 0.82 |
| K; meq.l^-1^ | 4.3 (4.1–4.6 [3.8–4.8]) | 4.3 (4.1–4.5 [3.8–4.4]) | 4.2 (4.0–4.6 [3.8–4.8]) | 0.56 |
| Creatinine; µmol.l^-1^ | 80 (66–95 [25–145]) | 80 (67–94 [45–140]) | 80 (66–98 [25–145]) | 0.83 |
| Urea; mmol.l^-1^ | 6.2 (4.6 – 7.5 [1.4–15]) | 6.2 (4.7–7.5 [1.4–15]) | 6 (4.6–7.5 [1.4–12]) | 0.14 |
| White blood count. x 10^9^.l^-1^ | 7.7 (6.2 – 8.8 [2.14–25.2]) | 7.7 (6.3–8.9 [4.21–25.2]) | 7.6 (5.9–8.7 [2.14–18.3]) | 0.64 |
| **Surgical specialty (%)** | | | | |
| Colorectal | 244 (20%) | 196 (20%) | 48 (20%) | 0.80 |
| Upper GI | 196 (17%) | 155 (17%) | 41 (17%) | 0.80 |
| Genito-urinary | 404 (34%) | 322 (34%) | 82 (34%) | 0.80 |
| Head and Neck | 253 (21%) | 201 (21%) | 52 (21%) | 0.80 |
| Thoracic | 43 (4%) | 33 (4%) | 10 (4%) | 0.80 |
| Others | 50 (4%) | 30 (4%) | 10 (4%) | 0.80 |
| **CPET values** | | | | |
| Systolic BP; mmHg | 131 (121–145 [96–189]) | 131 (121–145 [96–189]) | 132(121–146 [101–182]) | 0.68 |
| Diastolic BP; mmHg | 79 (70–87 [44–89]) | 79 (70–87 [44–89]) | 78 (71–86 [44–89]) | 0.23 |
| Mean BP; mmHg | 97 (88–105 [57–121]) | 97 (88–105 [57–121]) | 96 (88–105 [65–121]) | 0.33 |
| SpO_2_; % | 96 (96–97 [96–100]) | 96 (96–97 [96–100]) | 96 (96–97 [96–100]) | 0.56 |
| **CPET; baseline** | | | | |
| Heart rate; bpm | 82 (73–93) | 83 (73–93) | 82 (73– 93) | 0.61 |
| V̇O_2_.kg^-1^ ml.kg^-1^.min^-1^ | 3.79 (3.24–4.36) | 3.78 (3.23–4.38) | 3.81 (3.32–4.32) | 0.63 |
| RER | 0.82 (0.79–0.87) | 0.82 (0.79–0.87) | 0.82 (0.77–0.87) | 0.89 |
| VE.V̇CO_2_^-1^; ml.min^-1^ | 31.2 (29.3–33.6) | 31.4 (29.2–33.5) | 31.6 (29.4–33.9) | 0.38 |
| **CPET; anaerobic threshold** | | | | |
| Heart rate; bpm | 107 (95–118) | 106 (96–117) | 107 (94–120) | 0.28 |
| RER | 0.85 (0.81–0.89) | 0.85 (0.81–0.89) | 0.85 (0.80–0.89) | 0.10 |
| VE.V̇CO_2_^-1^; ml.min^-1^ | 33.2 (30.3–36.6) | 33.5 (30.2–36.5) | 33.8 (30.4–36.9) | 0.38 |
| **CPET – Peak** | | | | |
| Heart rate; bpm | 136 (121–152) | 136 (121–152) | 137 (12–152) | 0.58 |
| RER | 1.12 (1.03–1.2) | 1.12 (1.03–1.20) | 1.11 (1.04–1.20) | 0.70 |

P-POSSUM, Portsmouth-Physiological and Operative Severity Score for the enUmeration of Mortality and Morbidity; GI, gastro-intestinal; BP, blood pressure; V̇O_2_.kg^-1^, oxygen consumption per kilo; RER, respiratory equivalent ratio; VE.V̇CO_2_^-1^, ventilatory efficiency/carbon dioxide output.

# **Table S2.** Cox proportional hazards model analysing the relationship between various clinical features and 1-year mortality. Values are hazard ratios with 95% upper and lower coefficient.

| **Feature** | **Coef** | **Coef Lower 95%** | **Coef Upper 95%** | **P value** |
| --- | --- | --- | --- | --- |
| VO_2__Rest | 12.94 | 0.00 | 43707.88 | 0.54 |
| VCO_2__Rest | 4.12 | 0.00 | 32978.00 | 0.76 |
| ECG_ischaemia_rest | 3.58 | 0.55 | 23.36 | 0.18 |
| Peripheral_vascular_disease | 3.47 | 0.78 | 15.49 | 0.10 |
| RER_AT | 2.78 | 0.01 | 1255.01 | 0.74 |
| RER_VOP | 2.58 | 0.15 | 43.85 | 0.51 |
| MI | 2.43 | 1.12 | 5.24 | 0.02 |
| VCO_2__AT | 2.16 | 0.16 | 28.80 | 0.56 |
| CVA_or_TIA | 2.07 | 0.58 | 7.34 | 0.26 |
| Arthritis | 1.92 | 0.94 | 3.94 | 0.08 |
| Angina | 1.83 | 0.39 | 8.63 | 0.45 |
| VO_2__AT | 1.74 | 0.17 | 17.87 | 0.64 |
| Asthma | 1.67 | 0.57 | 4.87 | 0.35 |
| op_severity_SORT | 1.62 | 0.79 | 3.32 | 0.19 |
| VO_2__VOP | 1.54 | 0.47 | 5.04 | 0.48 |
| VCO_2__VOP | 1.45 | 0.57 | 3.66 | 0.44 |
| VE_VCO_2__VOP | 1.17 | 1.09 | 1.25 | 0.00 |
| VE_VCO_2__AT | 1.15 | 1.07 | 1.24 | 0.00 |
| VO_2__Kg_Rest | 1.13 | 0.67 | 1.92 | 0.65 |
| PetCO_2__Rest | 1.10 | 0.97 | 1.24 | 0.16 |
| Op_access | 1.09 | 0.86 | 1.37 | 0.48 |
| WBC | 1.08 | 0.98 | 1.19 | 0.12 |
| PetCO_2__VOP | 1.08 | 0.98 | 1.19 | 0.14 |
| SPO_2__Rest | 1.06 | 0.86 | 1.32 | 0.57 |
| Op_speciality | 1.06 | 0.94 | 1.20 | 0.36 |
| VO_2__Kg_AT | 1.05 | 0.87 | 1.26 | 0.64 |
| VO_2__HR_AT | 1.04 | 0.83 | 1.31 | 0.72 |
| VO_2__HR_VOP | 1.04 | 0.88 | 1.23 | 0.63 |
| PetO_2__AT | 1.03 | 0.95 | 1.11 | 0.48 |
| Hypertension | 1.03 | 0.51 | 2.06 | 0.94 |
| VE_AT | 1.02 | 0.94 | 1.10 | 0.65 |
| Age | 1.02 | 0.99 | 1.04 | 0.23 |
| HR_Rest | 1.02 | 0.99 | 1.05 | 0.27 |
| Diabetes | 1.01 | 0.43 | 2.41 | 0.97 |
| PetCO_2__AT | 1.01 | 0.91 | 1.12 | 0.85 |
| HR_VOP | 1.01 | 0.99 | 1.03 | 0.48 |
| Hb | 1.01 | 0.99 | 1.02 | 0.44 |
| Creatinine | 1.00 | 1.00 | 1.01 | 0.28 |
| HR_AT | 1.00 | 0.98 | 1.03 | 0.78 |
| WR_AT | 1.00 | 0.98 | 1.02 | 0.89 |
| VE_VCO_2__Rest | 1.00 | 0.94 | 1.07 | 0.97 |
| RR_Rest | 1.00 | 0.90 | 1.12 | 0.99 |
| BP_Sys_Rest | 1.00 | 0.98 | 1.02 | 0.98 |
| PetO_2__Rest | 1.00 | 0.92 | 1.09 | 0.95 |
| WR_VOP | 1.00 | 0.99 | 1.01 | 0.52 |
| VE_VO_2__VOP | 1.00 | 0.93 | 1.06 | 0.92 |
| PetO_2__VOP | 1.00 | 0.93 | 1.07 | 0.93 |
| RR_VOP | 1.00 | 0.93 | 1.06 | 0.87 |
| Weight | 0.99 | 0.96 | 1.03 | 0.72 |
| BP_Mean_Rest | 0.99 | 0.95 | 1.04 | 0.74 |
| VE_VOP | 0.99 | 0.96 | 1.02 | 0.53 |
| BP_Dia_Rest | 0.99 | 0.95 | 1.02 | 0.53 |
| VO_2__Kg_VOP | 0.99 | 0.90 | 1.09 | 0.79 |
| RR_AT | 0.98 | 0.90 | 1.07 | 0.68 |
| VE_VO_2__Rest | 0.98 | 0.91 | 1.06 | 0.64 |
| BMI | 0.97 | 0.89 | 1.07 | 0.57 |
| Urea | 0.96 | 0.87 | 1.05 | 0.37 |
| VE_VO2_AT | 0.95 | 0.86 | 1.05 | 0.33 |
| VE_Rest | 0.95 | 0.77 | 1.16 | 0.61 |
| ACE_inhibitor | 0.93 | 0.40 | 2.13 | 0.86 |
| VO_2__HR_Rest | 0.92 | 0.55 | 1.52 | 0.73 |
| ECG_ischaemia_exercise | 0.91 | 0.41 | 2.03 | 0.82 |
| MET | 0.90 | 0.64 | 1.27 | 0.53 |
| Cardiac_failure | 0.85 | 0.33 | 2.24 | 0.75 |
| Pulmonary_embolism | 0.85 | 0.12 | 5.83 | 0.87 |
| Beta_blocker | 0.84 | 0.23 | 3.03 | 0.79 |
| COPD | 0.82 | 0.26 | 2.61 | 0.73 |
| ASA | 0.75 | 0.48 | 1.18 | 0.21 |
| Gender | 0.75 | 0.34 | 1.65 | 0.47 |
| VT_AT | 0.72 | 0.19 | 2.73 | 0.63 |
| VT_VOP | 0.68 | 0.26 | 1.80 | 0.44 |
| Smoking | 0.67 | 0.36 | 1.25 | 0.20 |
| Statins | 0.58 | 0.28 | 1.22 | 0.15 |
| Height | 0.50 | 0.01 | 35.95 | 0.75 |
| Coronary_stent | 0.46 | 0.10 | 2.17 | 0.33 |
| Pulmonary_fibrosis | 0.41 | 0.00 | 248.61 | 0.79 |
| VT_Rest | 0.36 | 0.03 | 3.93 | 0.41 |
| Nitrates | 0.35 | 0.04 | 3.14 | 0.35 |
| RER_Rest | 0.05 | 0.00 | 10.55 | 0.27 |

# VO_2_, oxygen consumption; RER, respiratory exchange ratio; ECG, electrocardiogram; OP, operative; AT, anaerobic threshold; VE, ventilation; V̇CO_2_, carbon dioxide output; HR, heart rate; SpO_2_, oxygen saturation; RR, respiratory rate; BP, blood pressure; ACE, angiotensin-converting enzyme; WR, work rate; Hb, haemoglobin; BMI, body mass index; COPD, chronic obstructive pulmonary disease; MET, metabolic equivalent; VT, ventilatory threshold; VF, ventilatory frequency.

**Table S3.** Performance of different machine learning models, including logistic regression (LR), decision trees (DT); Extreme Gradient Boosting (XGBoost); AdaBoost (ADA); support vector machines (SVM); k-nearest neighbors (KNN); and a Multi-Objective Symbolic Regression (MOSR); on fitness dataset; clinical dataset and full dataset. Performance metrics are presented for both test and training sets and include area under the curve (AUC); accuracy; F1 score; precision (also known as positive predictive value or PPV); sensitivity; specificity; and negative predictive value (NPV). Values are mean (SD) or not available (na).

|  |  | --------------------------------------------------------------------- FITNESS Dataset ---------------------------------------------------------------- | | | | | | |
| --- | --- | --- | --- | --- | --- | --- | --- | --- |
| Test set |  | *AUC* | *Accuracy* | *F1 SCORE* | *Precision/PPV* | *Sensitivity* | *Specificity* | *NPV* |
|  | **LR** | 0.784 (0.012) | 0.914 (0.005) | 0.0 (0.000) | 1.0 (0.000) | 0.0 (0.000) | na (na) | 0.0 (0.000) |
|  | **DT** | 0.551 (0.013) | 0.885 (0.006) | 0.18 (0.028) | 0.228 (0.030) | 0.149 (0.028) | 0.228 (0.030) | 0.149 (0.028) |
|  | **RF** | 0.754 (0.027) | 0.918 (0.008) | 0.0 (0.000) | 0.0 (0.000) | 0.0 (0.000) | na (na) | 0.0 (0.000) |
|  | **XGBoost** | 0.812 (0.010) | 0.923 (0.005) | 0.179 (0.039) | 1.0 (0.000) | 0.099 (0.023) | 1.0 (0.000) | 0.099 (0.023) |
|  | **ADA** | 0.692 (0.024) | 0.929 (0.006) | 0.271 (0.039) | 1.0 (0.000) | 0.157 (0.026) | 1.0 (0.000) | 0.157 (0.026) |
|  | **SVM** | 0.668 (0.017) | 0.845 (0.005) | 0.317 (0.022) | 0.243 (0.017) | 0.457 (0.037) | 0.243 (0.017) | 0.457 (0.037) |
|  | **KNN** | 0.552 (0.021) | 0.911 (0.005) | 0.0 (0.000) | 1.0 (0.000) | 0.0 (0.000) | 0.0 (0.000) | 0.0 (0.000) |
|  | **MOSR** | 0.793 (0.013) | 0.856 (0.004) | 0.343 (0.021) | 0.278 (0.018) | 0.447 (0.032) | 0.278 (0.018) | 0.447 (0.032) |
| Training Set |  | *AUC* | *Accuracy* | *F1 SCORE* | *Precision/PPV* | *Sensitivity* | *Specificity* | *NPV* |
|  | **LR** | 0.881 (0.006) | 0.949 (0.002 | 0.431 (0.027) | 0.785 (0.031) | 0.297 (0.022 | 0.785 (0.031) | 0.297 (0.022) |
|  | **DT** | 1.0 (0.000) | 1.0 (0.000) | 1.0 (0.000) | 1.0 (0.000) | 1.0 (0.000) | 1.0 (0.000) | 1.0 (0.000) |
|  | **RF** | 1.0 (0.000) | 1.0 (0.000) | 1.0 (0.000) | 1.0 (0.000) | 1.0 (0.000) | 1.0 (0.000) | 1.0 (0.000) |
|  | **XGBoost** | 1.0 (0.000) | 1.0 (0.000) | 1.0 (0.000) | 1.0 (0.000) | 1.0 (0.000) | 1.0 (0.000) | 1.0 (0.000) |
|  | **ADA** | 0.998 (0.000) | 0.983 (0.001) | 0.851 (0.012) | 0.977 (0.001) | 0.754 (0.018) | 0.977 (0.001) | 0.754 (0.018) |
|  | **SVM** | 0.754 (0.008) | 0.853 (0.003) | 0.362 (0.012) | 0.252 (0.010) | 0.641 (0.017) | 0.252 (0.010) | 0.641 (0.017) |
|  | **KNN** | 0.904 (0.002) | 0.936 (0.004) | 0.056 (0.019) | 1.0 (0.000) | 0.029 (0.010) | 1.0 (0.000) | 0.029 (0.010) |
|  | **MOSR** | 0.889 (0.010) | 0.895 (0.003) | 0.472 (0.015) | 0.351 (0.017) | 0.724 (0.024) | 0.351 (0.017) | 0.724 (0.024) |
|  |  | --------------------------------------------------------------------------- **Clinical Dataset** ------------------------------------------------------------------------ | | | | | | |
| Test Set |  | *AUC* | *Accuracy* | *F1 SCORE* | *Precision/PPV* | *Sensitivity* | *Specificity* | *NPV* |
|  | **LR** | 0.63 (0.019) | 0.914 (0.005) | 0.0 (0.000) | 1.0 (0.000) | 0.0 (0.000) | na (na) | 0.0 (0.000) |
|  | **DT** | 0.495 (0.011) | 0.824 (0.006) | 0.087 (0.019) | 0.079 (0.018) | 0.099 (0.022) | 0.079 (0.018) | 0.099 (0.022) |
|  | **RF** | 0.761 (0.020) | 0.918 (0.008) | 0.0 (0.000) | 1.0 (0.000) | 0.0 (0.000) | na (na) | 0.0 (0.000) |
|  | **XGBoost** | 0.666 (0.027) | 0.909 (0.005) | 0.0 (0.000) | 1.0 (0.000) | 0.0 (0.000) | 0.0 (0.000) | 0.0 (0.000) |
|  | **ADA** | 0.525 (0.012) | 0.894 (0.008) | 0.082 (0.006) | 0.153 (0.018) | 0.056 (0.004) | 0.153 (0.018) | 0.056 (0.004) |
|  | **SVM** | 0.495 (0.017) | 0.742 (0.011) | 0.11 (0.016) | 0.076 (0.011) | 0.203 (0.031) | 0.076 (0.011) | 0.203 (0.031) |
|  | **KNN** | 0.492 (0.012) | 0.911 (0.005) | 0.0 (0.000) | 1.0 (0.000) | 0.0 (0.000) | 0.0 (0.000) | 0.0 (0.000) |
|  | **MOSR** | 0.594 (0.026) | 0.806 (0.007) | 0.21 (0.022) | 0.159 (0.016) | 0.308 (0.038) | 0.159 (0.016) | 0.308 (0.038) |
| Training Set |  | *AUC* | *Accuracy* | *F1 SCORE* | *Precision/PPV* | *Sensitivity* | *Specificity* | *NPV* |
|  | **LR** | 0.761 (0.017) | 0.938 (0.002) | 0.148 (0.012) | 0.707 ( 0.048) | 0.083 (0.007) | 0.707 (0.048) | 0.083 (0.007) |
|  | **DT** | 1.0 (0.000) | 1.0 (0.000) | 1.0 (0.000) | 1.0 (0.000) | 1.0 (0.000) | 1.0 (0.000) | 1.0 (0.000) |
|  | **RF** | 1.0 (0.000) | 1.0 (0.000) | 1.0 (0.000) | 1.0 (0.000) | 1.0 (0.000) | 1.0 (0.000) | 1.0 (0.000) |
|  | **XGBoost** | 1.0 (0.000) | 1.0 (0.000) | 1.0 (0.000) | 1.0 (0.000) | 1.0 (0.000) | 1.0 (0.000) | 1.0 (0.000) |
|  | **ADA** | 0.947 (0.003) | 0.942 (0.001) | 0.22 (0.018) | 0.874 (0.010) | 0.126 (0.011) | 0.874 (0.010) | 0.126 (0.011) |
|  | **SVM** | 0.529 (0.007) | 0.791 (0.005) | 0.124 (0.007) | 0.085 (0.006) | 0.228 (0.013) | 0.085 (0.006) | 0.228 (0.013) |
|  | **KNN** | 0.9 (0.003) | 0.936 (0.004) | 0.035 (0.002) | 1.0 (0.000) | 0.018 (0.001) | 1.0 (0.000) | 0.018 (0.001) |
|  | **MOSR** | 0.743 (0.011) | 0.841 (0.003) | 0.262 (0.012) | 0.188 (0.011) | 0.434 (0.015) | 0.188 (0.011) | 0.434 (0.015) |
|  |  | -------------------------------------------------------------------------- **Full Dataset** --------------------------------------------------------------------- | | | | | | |
| Test Set |  | *AUC* | *Accuracy* | *F1 SCORE* | *Precision/PPV* | *Sensitivity* | *Specificity* | *NPV* |
|  | **LR** | 0.759 (0.021) | 0.91 (0.006) | 0.226 (0.026) | 0.438 (0.049) | 0.153 (0.019) | 0.438 (0.049) | 0.153 (0.019) |
|  | **DT** | 0.62 (0.013) | 0.884 (0.006) | 0.307 (0.026) | 0.315 (0.041) | 0.303 (0.024) | 0.315 (0.041) | 0.303 (0.024) |
|  | **RF** | 0.801 (0.017) | 0.918 (0.008) | 0.0 (0.000) | 1.0 (0.000) | 0.0 (0.000) | na (na) | 0.0 (0.000) |
|  | **XGBoost** | 0.817 (0.018) | 0.926 (0.004) | 0.248 (0.038) | 1.0 (0.000) | 0.142 (0.025) | 1.0 (0.000) | 0.142 (0.025) |
|  | **ADA** | 0.732 (0.019) | 0.93 (0.007) | 0.386 (0.040) | 0.724 (0.059) | 0.264 (0.031) | 0.724 (0.059) | 0.264 (0.031) |
|  | **SVM** | 0.604 (0.021) | 0.691 (0.009) | 0.203 (0.016) | 0.128 (0.010) | 0.5 (0.043) | 0.128 (0.010) | 0.5 (0.043) |
|  | **KNN** | 0.481 (0.023) | 0.911 (0.005) | 0.0 (0.000) | 1.0 (0.000) | 0.0 (0.000) | 0.0 (0.000) | 0.0 (0.000) |
|  | **MOSR** | 0.939 (0.014) | 0.905 (0.007) | 0.712 (0.025) | 0.566 (0.023) | 0.911 (0.037) | 0.566 (0.023) | 0.911 (0.037) |
| Training Set |  | *AUC* | *Accuracy* | *F1 SCORE* | *Precision/PPV* | *Sensitivity* | *Specificity* | *NPV* |
|  | **LR** | 0.925 (0.005) | 0.953 ( 0.002) | 0.517 (0.017) | 0.795 ( 0.019) | 0.384 (0.018 | 0.795 (0.019) | 0.384 (0.018) |
|  | **DT** | 1.0 (0.000) | 1.0 (0.000) | 1.0 (0.000) | 1.0 (0.000) | 1.0 (0.000) | 1.0 (0.000) | 1.0 (0.000) |
|  | **RF** | 1.0 (0.000) | 1.0 (0.000) | 1.0 (0.000) | 1.0 (0.000) | 1.0 (0.000) | 1.0 (0.000) | 1.0 (0.000) |
|  | **XGBoost** | 1.0 (0.000) | 1.0 (0.000) | 1.0 (0.000) | 1.0 (0.000) | 1.0 (0.000) | 1.0 (0.000) | 1.0 (0.000) |
|  | **ADA** | 0.999 (0.000) | 0.983 (0.001) | 0.847 (0.013) | 1.0 (0.000) | 0.734 (0.020) | 1.0 (0.000) | 0.734 (0.020) |
|  | **SVM** | 0.683 (0.008) | 0.718 (0.006) | 0.229 (0.008) | 0.139 (0.006) | 0.643 (0.013) | 0.139 (0.006) | 0.643 (0.013) |
|  | **KNN** | 0.92 (0.003) | 0.942 (0.004) | 0.241 (0.021) | 0.805 (0.035) | 0.142 (0.014) | 0.805 (0.035) | 0.142 (0.014) |
|  | **MOSR** | 0.979 (0.001) | 0.921 (0.003) | 0.725 (0.014) | 0.55 (0.016) | 0.968 (0.007) | 0.55 (0.016) | 0.968 (0.007) |
