# Supplementary material for: Mortality prediction after major surgery in a mixed population through machine learning: a multi‐objective symbolic regression approach
Source: Anaesthesia. 2025 Jan 8;80(5):551–60. doi: 10.1111/anae.16538 (PMC7617356; doi:10.1111/anae.16538)
Supplement: Supplementary file 3 — Appendix S1. Model information. [file ANAE-80-551-s002.docx]

**Appendix S1.** Model information.

1. **Best performing model from MOSR with CFR dataset**

(0.05133353420624052 + (0.10773037504016572 * (0.1351472316719875 + ((0.019179032037781332 * VCO2_Rest) + ((-0.0013063991653400032 * VO2_HR_AT) + ((0.0007228267429418415 * HR_Rest) + ((0.000779124830564071 * VO2_Kg_Rest) + ((0.0638073479127414 * (1.1446089518569504 ** (RER_AT + ((0.9294175228539225 * (VE_VCO2_VOP ** 0.14361952671783987)) + ((-0.4758244802936393 * VE_VO2_AT) + ((-0.04718841465897728 * VE_VOP) + ((0.012496298073365528 * ((VE_VCO2_VOP + (-0.11180096050728633 * PetCO2_Rest)) * (VCO2_VOP + (VE_VCO2_AT + ((3.009415482454716 * PetCO2_VOP) + (-0.6268907104102416 * VE_VCO2_Rest)))))) + (0.1705003702505623 * (HR_Rest * RER_AT))))))))) + ((-0.00023572235969615448 * ((RER_AT + VT_VOP) ** 0.5854323924461247)) + ((0.021967825682468765 * (BP_Sys_Rest * ECG_ischaemia_rest)) + (0.1027745357186381 * ((1.2068607656566968 + VE_VO2_Rest) * (-23.348267320724027 + ((4.220989725036166 * RER_AT) + ((1.5857334488527037 * ECG_ischaemia_rest) + ((0.10317825444321034 * (HR_Rest * RER_AT)) + ((0.00015521320849333105 * ((VE_VCO2_VOP + (4.226756791805438 * PetCO2_Rest)) * (1.4010996248044114 + (VO2_Kg_Rest + (WR_VOP + (9.98570250453383 * VE_VCO2_Rest)))))) + ((-0.0026077127528636715 * ((VE_VCO2_VOP + (0.43972892096927557 * PetCO2_Rest)) * (HR_Rest + (VCO2_VOP + ((-1.2144125864636306 * VE_VCO2_Rest) + (PetCO2_VOP * RER_Rest)))))) + (-2.616644291516109 * (VO2_Rest * VT_Rest))))))))))))))))))))

**Features:** HR_Rest, VO2/HR_Rest, VE_Rest, RR_Rest, VO2_Rest, VO2/Kg_Rest, VCO2_Rest, RER_Rest, VE/VO2_Rest, VE/VCO2_Rest, VT_Rest, PetO2_Rest, PetCO2_Rest, SPO2_Rest, BP_Sys_Rest, BP_Dia_Rest, BP_Mean_Rest, HR_AT, VO2/HR_AT, VE_AT, RR_AT, VO2_AT, VO2/Kg_AT, VCO2_AT, RER_AT, VE/VO2_AT, VE/VCO2_AT, WR_AT, VT_AT, PetO2_AT, PetCO2_AT, HR_VOP, VO2/HR_VOP, VE_VOP, RR_VOP, VO2_VOP, VO2/Kg_VOP, VCO2_VOP, RER_VOP, VE/VO2_VOP, VE/VCO2_VOP, WR_VOP, VT_VOP, PetO2_VOP, PetCO2_VOP, MET, ECG_ischaemia_rest, ECG_ischaemia_exercise.

1. **Best performing model from MOSR with CFR dataset**

(0.2916530447446875 + (-0.41461225237956323 * (-0.7184849031432174 + ((1.7298151303073632e-07 * (1.0000000150919994 / asa)) + ((0.4906655315627293 * smoking) + ((-0.20680818770801737 * asa) + ((-0.007706851201391733 * ((cardiac_failure * exp(ace_inhibitor)) ** 6.632742418920191)) + ((0.4123495971120942 * (urea * (-9.4466215446674 + ((5.476274235649943 * potassium) + ((-0.14183997347478008 * hypertension) + ((-0.6530466581420745 * log((exp(potassium) ** potassium))) + ((-0.4225702460201632 * op_severity_SORT) + ((COPD * smoking) + ((-0.034865629217378166 * (MI * (8.460024837782225 + diabetes))) + (0.03043583397954037 * (number_smoked * ((-0.589778443891734 + statins) * log(potassium))))))))))))) + ((7.705793361318474 * (coronary_stent * (hypertension ** 0.8556752257410918))) + (0.016047086413954068 * (asa * duke_score)))))))))))

**Features:** sex, age, height, weight, BMI, op_severity_SORT, op_access, op_speciality, MI, angina, coronary_stent, CABG, hypertension, cardiac_failure, peripheral_vascular_disease, CVA_or_TIA, diabetes, COPD, asthma, pulmonary_embolis, pulmonary_fibrosis, smoking, number_smoked, years_smoked, pack_years, arthritis, beta_blocker, nitrates, ace_inhibitor, statins, ncepod, asa, duke_score, creatinine, hb, sodium, potassium, urea, wcc.

1. **Best performing model from MOSR with full dataset**

(0.9283066644759719 + (0.33558293372800585 * (0.9307358935128123 + (CVA_or_TIA + (0.05433912262007312 * (PetO2_Rest * (-2.058269075967044 + ((4.266463775274639 * (VE_Rest ** (-35.009133336095616 + ((0.7567062490252787 * VT_VOP) + ((3.841820371862717 * RER_AT) + ((0.3184675679905378 * SPO2_Rest) + ((2.7717435866581797 * log(VE_VCO2_AT)) + ((0.16952523190991609 * op_access) + ((0.16985271203083768 * asa) + ((0.02919456996026764 * HR_Rest) + ((0.11769707996216218 * VT_AT) + ((0.22036815305302138 * COPD) + ((0.24197749749659594 * VE_VCO2_VOP) + ((-4.115925845086925 * coronary_stent) + ((-0.16548919963455938 * PetO2_Rest) + ((-0.16924369479944934 * VE_VO2_VOP) + (-0.0433483250620599 * (RR_Rest * peripheral_vascular_disease)))))))))))))))))) + ((0.6719755488358315 * (1.2307530852679749 ** (-45.66299838255008 + ((5.2924354367543724 * log(VE_VCO2_AT)) + ((0.007264491516315055 * PetO2_Rest) + ((0.6576966852082733 * arthritis) + ((0.09272920256684015 * VE_VCO2_VOP) + ((10.434158682203327 * RER_AT) + ((1.7966679823191978 * MI) + ((0.04711696622184297 * SPO2_Rest) + ((-0.09630752713504853 * HR_Rest) + ((-0.6219217782791184 * op_access) + ((-0.2622079848444287 * VT_VOP) + ((-0.27133113217408295 * VE_VO2_VOP) + ((-0.5716440485250398 * asa) + ((-1.2404315719918781 * VT_AT) + ((-1.411859231677732 * coronary_stent) + ((-1.4061197843058617 * COPD) + ((0.28703964511471003 * (RR_Rest * peripheral_vascular_disease)) + (-0.08168869724610851 * (VE_VO2_AT * (0.9999917267066163 / VO2_AT)))))))))))))))))))))) + ((0.01382793716640899 * (1.0534371899781594 ** (-38.474250753405855 + ((0.3208279570857723 * VT_AT) + ((3.18897060020776 * log(VE_VCO2_AT)) + ((0.06257406954922334 * RR_Rest) + ((0.34345401895299815 * VE_VCO2_VOP) + ((1.6000041184481424 * arthritis) + ((7.61312231850043 * RER_AT) + ((0.19534072672312935 * SPO2_Rest) + ((0.3153101399599752 * VT_VOP) + ((0.03112573107804196 * asa) + ((-1.0499380075976248 * coronary_stent) + ((-0.02635358255888334 * op_access) + ((-1.3565059546206961 * COPD) + ((-0.4093437068422539 * VE_VO2_VOP) + ((-0.219667461546001 * PetO2_Rest) + (-0.08599613233331431 * (HR_Rest * RER_VOP))))))))))))))))))) + ((0.09105232336124425 * wcc) + ((1.060617781363443 * (1.2475771444261878 ** (-45.623091575629296 + ((0.7216642093224814 * arthritis) + ((10.60208890638064 * RER_AT) + ((5.641623097181158 * log(VE_VCO2_AT)) + ((0.17022706523011877 * SPO2_Rest) + ((0.02472671570547256 * HR_Rest) + ((0.15207418979609325 * PetO2_Rest) + ((0.04744392850926266 * VE_VCO2_VOP) + ((1.568101303068128 * MI) + ((-0.06449044878054605 * VE_Rest) + ((-0.2803767180683207 * VE_VO2_VOP) + ((-0.5757610017751317 * op_access) + ((-0.5301692334869832 * asa) + ((-1.0108747197061234 * VT_AT) + ((-1.701060546951224 * COPD) + ((-0.16156125786974776 * VT_VOP) + ((0.3474999905546033 * (RR_Rest * peripheral_vascular_disease)) + (-0.09246954247712741 * (VE_VO2_AT * (1.000322926792997 / VO2_AT)))))))))))))))))))))) + ((0.11176962946430034 * (VE_AT * (-0.42999453170927876 + (0.6676490616988088 * ((VE_VCO2_AT ** 3.6929825676179653) * ((-35.53179655435609 + ((0.15076387956095139 * VT_AT) + ((0.2990095310080925 * SPO2_Rest) + ((0.9905364595957302 * VT_VOP) + ((0.04797672150047858 * op_access) + ((1.6849335480044298 * log(VE_VCO2_AT)) + ((0.5774583394524585 * VE_VCO2_VOP) + ((0.09141504534117899 * VE_VO2_VOP) + ((7.205296845519295 * RER_AT) + ((0.32143208611809787 * asa) + ((0.03470268671247951 * HR_Rest) + ((-0.31779426109753584 * PetO2_Rest) + ((-1.114098739943835 * coronary_stent) + ((-1.651203264797791 * COPD) + (0.33985889634154764 * (RR_Rest * peripheral_vascular_disease)))))))))))))))) * exp(((6.9481290453664935 * RER_AT) + ((0.1636987784765668 * HR_Rest) + ((1.2962027251877615 * arthritis) + ((-0.0798684155928878 * PetO2_Rest) + ((-1.4064721577433261 * asa) + ((-0.3553328810586064 * VE_VO2_VOP) + ((-0.7713770873368276 * coronary_stent) + (-0.41261844362477423 * VE_VCO2_VOP))))))))))))))) + ((0.6282951060919124 * ((VE_VCO2_AT ** 3.4339188207091813) * ((-36.00554305238255 + ((1.3312107330692728 * VCO2_VOP) + ((4.579567767070361 * RER_AT) + ((0.005115389445357043 * HR_Rest) + ((0.6967175063338192 * VE_VCO2_VOP) + ((0.21698929428160663 * SPO2_Rest) + ((0.4463670769795219 * asa) + ((0.22258066814671626 * op_access) + ((-0.08496010825981727 * PetO2_Rest) + ((-0.7059916914876796 * COPD) + ((-0.10447265871322216 * VE_VO2_VOP) + ((-0.7853203041987914 * log(VE_VCO2_AT)) + ((-0.44141263279738924 * VT_VOP) + ((-2.7568298164273317 * coronary_stent) + (-1.2213235516518384 * (RR_Rest * peripheral_vascular_disease)))))))))))))))) * exp(((2.5316957078140345 * arthritis) + ((2.7623655066996946 * ECG_ischaemia_rest) + ((0.08245636000992398 * VE_VO2_VOP) + ((0.7709717582291313 * coronary_stent) + ((0.04596281686530606 * RR_Rest) + ((-0.7873445448126613 * asa) + ((-0.3240275004407378 * VE_VCO2_VOP) + (-0.04084785703838275 * PetO2_Rest)))))))))))) + (0.34125811615998736 * ((VE_VCO2_AT ** 3.5894059925958546) * ((-36.012306148774684 + ((0.303406287619158 * SPO2_Rest) + ((0.8519225015405713 * op_access) + ((4.592567848379204 * RER_AT) + ((0.29345743398718105 * VT_VOP) + ((0.6509514344600934 * asa) + ((0.06203181482514852 * HR_Rest) + ((1.0252884479443967 * VCO2_VOP) + ((0.4878725444270376 * VE_VCO2_VOP) + ((-0.16002077831475395 * VE_VO2_VOP) + ((-0.8423939593241278 * COPD) + ((-0.14426994435750012 * PetO2_Rest) + ((-1.0071785452635833 * log(VE_VCO2_AT)) + ((-2.4175425995898046 * coronary_stent) + (-1.169349174985846 * (RR_Rest * peripheral_vascular_disease)))))))))))))))) * exp(((0.14711145550327137 * coronary_stent) + ((0.19846347755067764 * RR_Rest) + ((2.306561784443449 * arthritis) + ((0.16723240104705175 * VE_VO2_VOP) + ((3.3339553563782474 * RER_AT) + ((-0.7135622569973444 * VE_VCO2_VOP) + ((-0.010040343579610034 * PetO2_Rest) + (-0.4527169993815749 * asa))))))))))))))))))))))))))
